# A Facile Method for the Preparation of Colored Bi_4_Ti_3_O_12−*x*_ Nanosheets with Enhanced Visible-Light Photocatalytic Hydrogen Evolution Activity

**DOI:** 10.3390/nano8040261

**Published:** 2018-04-21

**Authors:** Yizeng Zhang, Zhiwu Chen, Zhenya Lu

**Affiliations:** 1 College of Materials Science and Engineering, South China University of Technology, Guangzhou 510640, China; zyzdd286@163.com (Y.Z.); zhylu@scut.edu.cn (Z.L.); 2 State Key Laboratory of Pulp and Paper Engineering, South China University of Technology, Guangzhou 510640, China

**Keywords:** Bi_4_Ti_3_O_12_ nanosheets, photocatalytic hydrogen evolution, solid-state chemical reduction, oxygen vacancy

## Abstract

Bi_4_Ti_3_O_12−*x*_ nanosheet photocatalysts with abundant oxygen vacancies are fabricated by a facile solid-state chemical reduction method for the first time. This method is simple in operation, has short reaction time, and can be conducted at mild temperatures (300~400 °C). The electron paramagnetic resonance, thermogravimetric analysis, X-ray photoelectron spectrometer, and positron annihilation lifetime spectra results indicate that oxygen vacancies are produced in Bi_4_Ti_3_O_12−*x*_, and they can be adjusted by tuning the reduction reaction conditions. Control experiments show that the reduction time and temperature have great influences on the photocatalytic activities of Bi_4_Ti_3_O_12−*x*_. The optimal Bi_4_Ti_3_O_12−*x*_ is the sample undergoing the reduction treatment at 350 °C for 60 min and it affords a hydrogen evolution rate of 129 μmol·g^−1^·h^−1^ under visible-light irradiation, which is about 3.4 times that of the pristine Bi_4_Ti_3_O_12_. The Bi_4_Ti_3_O_12−*x*_ photocatalysts have good reusability and storage stability and can be used to decompose formaldehyde and formic acid for hydrogen production. The surface oxygen vacancies states result in the broadening of the valence band and the narrowing of the band gap. Such energy level structure variation helps promote the separation of photo-generated electron-hole pairs thus leading to enhancement in the visible-light photocatalytic hydrogen evolution. Meanwhile, the narrowing of the band gap leads to a broader visible light absorption of Bi_4_Ti_3_O_12−*x*_.

## 1. Introduction

The development of green energy has become one of the most prominent research fields. Among many energy sources, hydrogen gas has been considered one of the prime candidates for solving the emerging worldwide energy crisis, owing to the fact that it does not produce pollution and it has high energy density [1,2]. In recent years, hydrogen generation through photocatalytic water splitting utilizing solar energy has become an extremely active research area [2]. An ideal photocatalyst should be stable, non-toxic, easily available from nature, able to function under visible light, and highly efficient in separating photo-generated electron-hole pairs. So far, various types of photocatalysts, such as TiO_2_ [3], ZnO [4], and CdS [5], have been developed. However, none of the photocatalysts can meet all the above requirements simultaneously and most of them suffer from a narrow photo response wavelength range because of large band gaps, which lead to very low utilization efficiency of solar energy. Therefore, the exploration of novel, efficient visible-light photocatalysts for splitting water to produce H_2_ is of utmost importance [6,7].

Bismuth titanate (Bi_4_Ti_3_O_12_) is well-known as a ferroelectric agent that has a special Aurivillius architecture with unique, electro-optic-converting performance. The Aurivillius phases of Bi_4_Ti_3_O_12_ have structures that are intergrown layers of [Bi_2_O_2_]^2+^ alternating with perovskite-like [Bi_2_Ti_3_O_10_]^2−^ blocks [8,9,10]. The advantage of such a layered structure is its ability to efficiently assist in diffusion and separation of the electron-hole pairs generated by light irradiation. Such separation increases the lifetime of the associated charge carriers, thus improving the quantum efficiency of photo-degradation [8,9,11]. Moreover, the hybridized 6s^2^ of Bi^3+^ and O2p generate a new valence band, which reduces the band gap of Bi_4_Ti_3_O_12_ [8,9]. Since the photocatalytic activity of Bi_4_Ti_3_O_12_ for water splitting was reported by Kudo et al., its photocatalytic properties have been receiving immense attention [12]. Various types of Bi_4_Ti_3_O_12_ photocatalysts, such as nanofibers [8,11], particles [12,13], platelets [14], and films [15] have been developed for processes involving alternative energy development or destruction of different pollutants. However, these Bi_4_Ti_3_O_12_ nanomaterials are not very efficient as visible-light photocatalysts because of the high recombination rate of photo-induced electron-hole pairs [16,17,18]. A widely adopted methodology to overcome this recombination problem is to couple Bi_4_Ti_3_O_12_ with semiconductors having narrower band gaps, such as Bi_2_MoO_6_ [11], BiOI [16], Bi_2_Ti_2_O_7_ [17], BiOCl [18], Ag_3_PO_4_ [19], and g-C_3_N_4_ [20]. However, the synthesis of composite compounds generally requires sophisticated synthesis techniques. Additionally, such synthesis increases the possibilities of introducing thermodynamic and structural instability due to the additionally introduced layer. Lastly, electrons would go through a multi-step transport process in such layered structures, which may diminish the efficient charge separation. Thus, catalysts comprised of single-phase metal oxides are highly desirable because they can provide both reliable stability and efficient electron-hole separation [21]. It is therefore very important to explore facile and economic techniques to prepare a single-phase Bi_4_Ti_3_O_12_ photocatalyst with increased absorbance of visible light and a low carrier recombination rate.

It has been found recently that the oxygen vacancy defects in TiO_2_ [22,23], ZnO [24,25], and Fe_2_O_3_ [26] are able to enhance photocatalytic performance. In particular, surface oxygen vacancies can first capture photo-generated electrons and then promote the reaction between these electrons with the adsorbed species, thus effectively preventing the recombination of photo-generated electron-hole pairs and improving the photocatalytic performance [22,23,24,25,26]. Bulk oxygen vacancies, on the contrary, are recombination centers of photo-generated electron-hole pairs and will reduce the photocatalytic performance [25]. The surface and bulk oxygen vacancies play different roles in the photocatalytic reaction. However, the effect of oxygen vacancies on the photocatalytic activity of Bi_4_Ti_3_O_12_ has not been thoroughly explored. Therefore, the development of cost-effective synthesis procedures for the production of Bi_4_Ti_3_O_12−*x*_ with oxygen vacancies and the in-depth understanding of its catalytic behavior are of profound importance in order to realize fully the great potential of Bi_4_Ti_3_O_12−*x*_ in water splitting for H_2_ production.

We recently reported a sol-gel hydrothermal technique to prepare highly crystalline Bi_4_Ti_3_O_12_ nanosheets with enhanced catalytic activity towards the photodegradation of Rhodamine B, especially when comparing with the calcined sample [9]. In the present work, to improve the photocatalytic activity even further, a Bi_4_Ti_3_O_12_ nanosheet photocatalyst with oxygen vacancies (Bi_4_Ti_3_O_12−*x*_) was fabricated for the first time by the solid-state chemical reduction method using NaBH_4_ and Bi_4_Ti_3_O_12_ nanosheets. The reported methods on oxygen vacancy creation include heating the sample under an oxygen-deficient atmosphere (e.g., vacuum) or reducing conditions (e.g., H_2_) [25,26], chemical vapor deposition, high-energy particle (laser, electron, or Ar^+^) bombardment [27], combustion method [23], high pressure [28], high temperature aluminum vapor reduction [29], etc. For practical application, these strategies have a number of limitations, such as multiple steps, harsh synthesis conditions (high temperature (>500 °C) or high-pressure hydrogen (20 bar)), or expensive facilities. Compared with the above traditional methods, the solid-state chemical reduction method has many advantages, such as simple operation, moderate reaction temperature (350 °C), short reaction time (less than 80 min), simple equipment, etc. Using this method, several Bi_4_Ti_3_O_12−*x*_ samples with different reduction degrees and, as a consequence, with different colors (several shades of blue, as well as black) have been synthesized. The effects of reduction time and temperature on the visible-light photocatalytic properties of the as-prepared Bi_4_Ti_3_O_12−*x*_ nanosheets have been investigated systematically. The electron paramagnetic resonance (EPR), thermogravimetric analysis (TGA), and positron annihilation lifetime spectra (PALS) indicate that oxygen vacancies are produced on Bi_4_Ti_3_O_12_ nanosheets during the reduction process. It has been found that the aggregation of oxygen vacancies raises the valence band maximum (VBM), thus decreasing the band gap and extending the photo response wavelength range. Moreover, the energy level variation induced by oxygen vacancy can facilitate the separation efficiency of the photo-generated electron-hole pairs, which contributes significantly to the improvement of the photocatalytic performance of Bi_4_Ti_3_O_12−*x*_. In this paper, we also propose a mechanism for the decrease of the band gap of Bi_4_Ti_3_O_12−*x*_ and its photocatalytic activity improvement.

## 2. Experimental Section

### 2.1. Synthesis of the Bi_4_Ti_3_O_12−x_ Nanosheet Photocatalyst

Bi_4_Ti_3_O_12_ nanosheets were synthesized using a sol-gel hydrothermal technique reported in our previous work (see Supplementary Materials) [9]. Bi_4_Ti_3_O_12−*x*_ catalysts were prepared as follows: 4 g of the as-prepared Bi_4_Ti_3_O_12_ nanosheets and 1 g NaBH_4_ were ground together for 45 min. Then, the mixture was put into a quartz tube placed in a tubular furnace; the temperature of the furnace was controlled by a heating device. The mixture was calcined at 350 °C for 20~100 min or 300~400 °C for 60 min under a nitrogen atmosphere with a ramping up rate of 10 °C/min. After the completion of this solid-state chemical reduction process, the powders were allowed to cool down to ambient temperature. Then, the final product was filtered, rinsed with deionized water and ethanol, and then dried at 80 °C. The samples were marked as Bi_4_Ti_3_O_12−*x*_ (T, t), where T is the temperature and t is the time of the solid-state chemical reduction procedure.

### 2.2. Characterization

The crystalline structures of the samples were examined by an X-ray diffractometer (XRD, D/Max-3C, Rigaku Co., Tokyo, Japan) using Cu K*α* radiation (*λ* = 1.5418 Å). The chemical states and composition were examined using an X-ray photoelectron spectrometer (XPS, Axis uhru DCD, Manchester, UK) with a monochromatic Mg K*α* X-ray source. The morphology was characterized using a scanning electron microscopy (SEM, LEO 1530 VP, Zeiss, Oberkochen, Germany) with 20 kV of accelerating voltage, and the composition was examined using energy dispersive spectroscopy (EDS, Zeiss, Oberkochen, Germany) attached to the SEM. Transmission electron microscopy (TEM) and high-resolution transmission electron microscopy (HRTEM) were performed using JEOL-2011 instrument (JEOL, Tokyo, Japan). UV-Vis spectrophotometer (lambda 35, Perkin-Elmer, Shelton, WA, USA) was used to analyze the samples’ diffuse reflection spectra with BaSO_4_ as a reference. TGAs over a temperature range of 25–800 °C were conducted during progressive heating (10 °C/min) in an air atmosphere by a thermogravimetric analyzer (SDTA 851e, Mettler Toledo, Zurich, Switzerland). The EPR analysis was performed on an Endor spectrometer JEOL ES-ED3X (JEOL, Tokyo, Japan). The specific surface areas of the powders were determined using the Brunauer-Emmett-Teller (BET) method after cooling down the samples with liquid nitrogen. PALS were obtained by ORTEC-583 fast-slow coincident system.

Photocurrent and electrochemical impedance spectroscopy (EIS) measurements were conducted at frequencies between 1 × 10^−5^ and 100 kHz by a CHI 660 electrochemical instrument (CH Instruments, CH Instruments, Austin, TX, USA) in 0.5 M Na_2_SO_4_ electrolyte solution. A three-electrode cell system was implemented with ITO (Indium tin oxide)/Bi_4_Ti_3_O_12_ (or Bi_4_Ti_3_O_12−*x*_) as the working electrode, Pt wire as the counter electrode, and standard calomel electrode (SCE) as the reference. The true potentials were calculated in reference to the results from the SCE. The visible light source was a 300 W Xe lamp with a 400 nm cut-off filter. The photocatalyst photoelectric responses “on and off” were determined at 0.0 V. Moreover, the ITO/Bi_4_Ti_3_O_12_ (or Bi_4_Ti_3_O_12−*x*_) electrodes were produced using the following recipe: First, Bi_4_Ti_3_O_12_ (or Bi_4_Ti_3_O_12−*x*_) samples (5 mg) were mixed with ethyl alcohol (0.15 mL) and 5% Nafion DE 520 solution (0.35 mL). This solution was then homogenized under ultrasound agitation for 20 min. Uniform film electrodes were prepared by casting 0.1 mL of the Bi_4_Ti_3_O_12_ (or Bi_4_Ti_3_O_12−*x*_) slurry onto pre-cleaned ITO glass (<7 ohm/square). The ITO/Bi_4_Ti_3_O_12_ (or Bi_4_Ti_3_O_12−*x*_) electrodes were finalized by sintering at 100 °C for 2 h.

### 2.3. Photocatalytic Activity

Photocatalytic hydrogen evolution experiments were proceeded in a methanol-water mixture and performed in the outer quartz ampules attached to the airtight gas circulation system. In order to promote the dispersion of Bi_4_Ti_3_O_12_ or Bi_4_Ti_3_O_12−*x*_ in the methanol-water mixture, the optimal dispersing process was obtained through orthogonal tests. The Bi_4_Ti_3_O_12_ or Bi_4_Ti_3_O_12−*x*_ (0.25 g) was dispersed by ultrasound agitation (15 min) in the mixture of deionized water (DI water, 200 mL) and methanol (20 mL), followed by constant stirring for 30 min. To eliminate dissolved oxygen, the solution was purged with argon for 30 min. This step is necessary to minimize the recombination reaction between H_2_ and O_2_ during the water splitting reaction. It also improves the purity of the hydrogen and reduces explosion risks.

Upon finishing the preparation steps, the reactor was irradiated by a 300 W Xe lamp for 4 h. A cut-off filter for visible-light irradiation was used to obtain wavelengths above 400 nm. The total amount of hydrogen evolved was measured by a gas chromatographer GC-3240/TCD (Perfect Light, Beijing, China), which used Ar carrier gas and was directly connected to a gas-circulation line. The suspensions of the photocatalyst were magnetically stirred continuously during the photocatalytic hydrogen evolution. An average of five measurements was adopted to determine the yield. To study the reusability and repeatability, the same Bi_4_Ti_3_O_12−*x*_ photocatalyst was used three times, and the amount of hydrogen evolved was recorded. After each measurement, the catalyst was centrifugated, filtrated, washed by DI water, and dried at 100 °C for 3 h for the next measurement. To check the dependency of H_2_ production on the additives, the same experiments were also performed with formaldehyde and formic acid instead of methanol.

## 3. Results and Discussion

### 3.1. Morphology, Structure, and UV-Vis Spectra of Bi_4_Ti_3_O_12_ and Bi_4_Ti_3_O_12−x_

**Figure 1:**
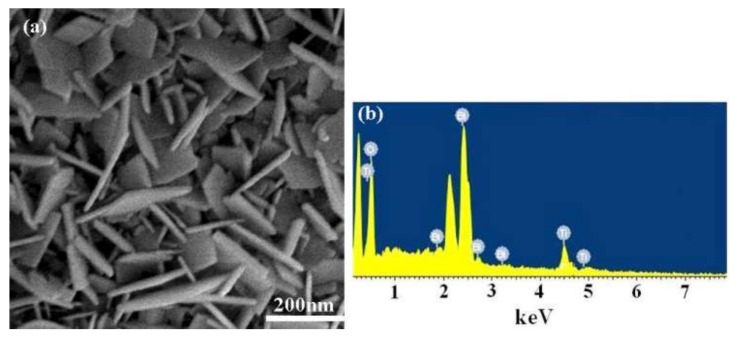
(**a**) SEM image; (**b**) energy dispersive spectroscopy (EDS) pattern of the pristine Bi_4_Ti_3_O_12_ powders synthesized using a sol-gel hydrothermal technique at 160 °C for 16 h.

The morphology and particle size distribution of a pristine Bi_4_Ti_3_O_12_ were scrutinized using SEM. As shown in Figure 1a, the Bi_4_Ti_3_O_12_ sample consists mostly of regular rectangular nanosheets with a narrow distribution of rim size and thin thickness. The average rim size of the rectangular nanosheets are about 100 and 150 nm, respectively, and thickness is about 20 nm. As shown in Figure 1b, only three elements of Bi, Ti, and O are observed with the EDS measurement. The ratio of the Bi, Ti, and O elements is 20.98:15.75:63.27, further demonstrating that the pure Bi_4_Ti_3_O_12_ was hydrothermally synthesized successfully at 160 °C.

**Figure 2:**
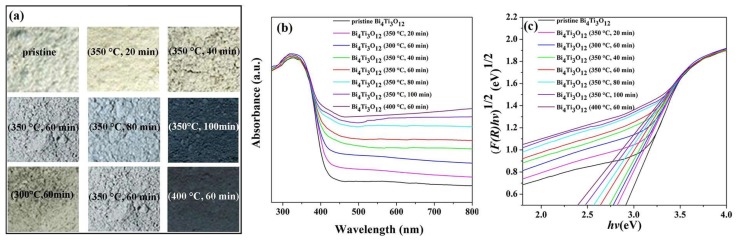
(**a**) photographs of pristine Bi_4_Ti_3_O_12_ and various colored Bi_4_Ti_3_O_12−*x*_; (**b**) UV-Vis absorption spectrum of the pristine Bi_4_Ti_3_O_12_ and various Bi_4_Ti_3_O_12−*x*_; (**c**) plot of the transformed Kubelka-Munk function (F(*R*∞)) versus the photon energy (h*v*) for various Bi_4_Ti_3_O_12−*x*_ and the pristine Bi_4_Ti_3_O_12_ nanosheets.

When the mixture of Bi_4_Ti_3_O_12_ nanosheets and NaBH_4_ were heated at 350 °C for 20~100 min and 300~400 °C for 60 min under nitrogen, NaBH_4_ decomposed and produced active hydrogen. It has been reported that the reduction ability of this active hydrogen is greater than that of H_2_ and other reducing agents at these temperature [23,30,31]. Hydrogen is a strong reducing agent capable of reacting fast and generating oxygen vacancies in Bi_4_Ti_3_O_12_ at relatively low temperatures, as well as maintaining the original shape of the Bi_4_Ti_3_O_12_ nanosheets at the same time. As shown in Figure 2a, the color of the Bi_4_Ti_3_O_12−*x*_ samples is changed clearly after the solid-state chemical reduction. When the mixture of Bi_4_Ti_3_O_12_ nanosheets and NaBH_4_ were heated at 350 °C, it could be seen that with the increased duration of the reaction, the colors of Bi_4_Ti_3_O_12−*x*_ changed from white-yellow to light blue, then to dark blue. Moreover, the colors of Bi_4_Ti_3_O_12−*x*_ also became darker with the increase in temperature. When the reaction temperature rises to 400 °C, black Bi_4_Ti_3_O_12−*x*_ can be synthesized in 60 min, suggesting that the solid-state chemical reduction process modifies the surface features of the Bi_4_Ti_3_O_12_ nanosheets. According to previous reports [32,33], it is highly possible that the color change could be caused by the formation of oxygen vacancies occurring during the reduction. The different colors of the Bi_4_Ti_3_O_12−*x*_ samples indicate that various reduction degrees of Bi_4_Ti_3_O_12−*x*_ can be obtained by adjusting the reaction time or temperature, which is helpful to understand the formation mechanism of reductive Bi_4_Ti_3_O_12_. Moreover, the XPS results show no residue of B and Na in Bi_4_Ti_3_O_12−*x*_ (see Figure S1 in Supplementary Materials), which means that the coproducts from NaBH_4_ during the solid-state chemical reduction method can be cleaned easily by washing with water and ethanol.

To understand the effect of the solid-state chemical reduction treatment on the optical absorption property of the photocatalyst, the UV-Vis diffuse reflectance spectra of various Bi_4_Ti_3_O_12−*x*_ and the pristine Bi_4_Ti_3_O_12_ nanosheets were examined, as shown in Figure 2b. It can be seen that the pristine Bi_4_Ti_3_O_12_ shows a typical spectrum with an absorption edge at about 420 nm. Compared with the pristine Bi_4_Ti_3_O_12_ sample, the absorption edge of Bi_4_Ti_3_O_12−*x*_ exhibits a clear red shift to higher wavelengths. In addition, the absorbance intensity of Bi_4_Ti_3_O_12−*x*_ in the range of 400–800 nm increases with both reduction time and temperature, which agrees with the color variation in the samples. The red shift of the absorption edge and the enhanced absorbance intensity of Bi_4_Ti_3_O_12−*x*_ are probably because of the different surface conditions of different samples. Surface defects, such as oxygen vacancies, generally affect the atomic structure of a photocatalyst and its surface states, which play a very important role in the overall photocatalytic activity [33,34].

The band gap can be calculated using the UV-Vis data from the following equation [8]:

*α*h*v* = A(h*v* − *E*_g_)^n/2^ (1)

where *α* is an absorption coefficient, h is Planck’s constant, *v* is light frequency, *E*_g_ is a band gap value, and A is a constant. The absorption behavior of Bi_4_Ti_3_O_12_ demonstrates indirect transition between bands; therefore, the value of n equal to 4 is used [8,35]. The value of the band gap is estimated by extrapolating the linear part of the (*α*h*v*)^1/2^ versus (h*v*) plot at *α* = 0. Normally, the collected UV-Vis diffuse reflectance spectra can be converted into Kubelka-Munk function F(*R*_∞_) based on the relationship shown in Equations (2) and (3) [36]:

Abs = −log *R*_∞_ (2)

F(*R*_∞_) = (1 − *R*_∞_)^2^/2*R*_∞_ = *α* (3)

where Abs is absorbance, and *R*_∞_ is reflectance. Therefore, Equation (1) can also be written as follows:

[F(*R*_∞_) h*ν*] = A(h*v* − *E*_g_)^n/2^ (4)

In addition, h*v =* hc*/λ* ≈ 1241/*λ* (eV). Figure 2c shows the plot of the transformed Kubelka-Munk function versus the photon energy for various Bi_4_Ti_3_O_12−*x*_ and the pristine Bi_4_Ti_3_O_12_ nanosheets. The energy of the band gap values is obtained by extrapolating the linear part of [F(*R*_∞_)h*ν*]^1/2^ versus h*v* plot at F(*R*_∞_) = 0. The band gap of the pristine Bi_4_Ti_3_O_12_ and various Bi_4_Ti_3_O_12−*x*_ samples are shown in Table 1. It is clear that the Bi_4_Ti_3_O_12−*x*_ samples show a decreased band gap value when compared with the pristine Bi_4_Ti_3_O_12_. Furthermore, it is also clear that the band gap of Bi_4_Ti_3_O_12−*x*_ decreases as reaction time and temperature increase.

**Figure 3:**
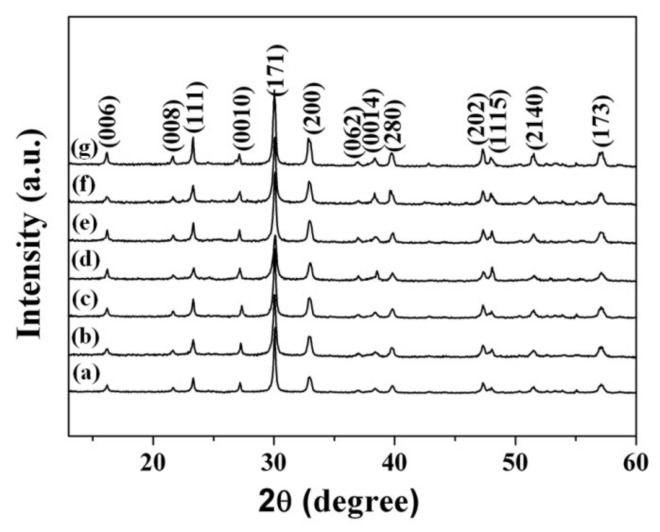
X-ray diffraction patterns of (a) the pristine Bi_4_Ti_3_O_12_; (b) Bi_4_Ti_3_O_12−*x*_ (350 °C, 20 min); (c) Bi_4_Ti_3_O_12−*x*_ (350 °C, 40 min); (d) Bi_4_Ti_3_O_12−*x*_ (350 °C, 60 min); (e) Bi_4_Ti_3_O_12−*x*_ (350 °C, 80 min); (f) Bi_4_Ti_3_O_12−*x*_ (350 °C, 100 min); and (g) Bi_4_Ti_3_O_12−*x*_ (400 °C, 60 min).

XRD analyses were performed to characterize the changes of the crystalline structures of the Bi_4_Ti_3_O_12−*x*_ samples. Figure 3 shows a comparison of XRD patterns of the pristine Bi_4_Ti_3_O_12_ and various Bi_4_Ti_3_O_12−*x*_ samples after they were treated at 350 °C for 20~100 min and at 400 °C for 60 min. No impurities can be seen for the Bi_4_Ti_3_O_12−*x*_ samples, indicating that the reduction process has no effect on the crystal structure. The diffraction peaks suggest that Bi_4_Ti_3_O_12−*x*_ samples have a high degree of crystalline similarity to Bi_4_Ti_3_O_12_. However, new peaks appear in the XRD patterns of the samples treated for a longer reaction time (350 °C for 120 min) or at a higher temperature (400 °C for 80 min) (Figure S2). We could not match these new peaks to any known powder diffraction file (PDF); thus, it is possible that new phases have been formed.

### 3.2. Photocatalytic Performance and Stability

**Figure 4:**
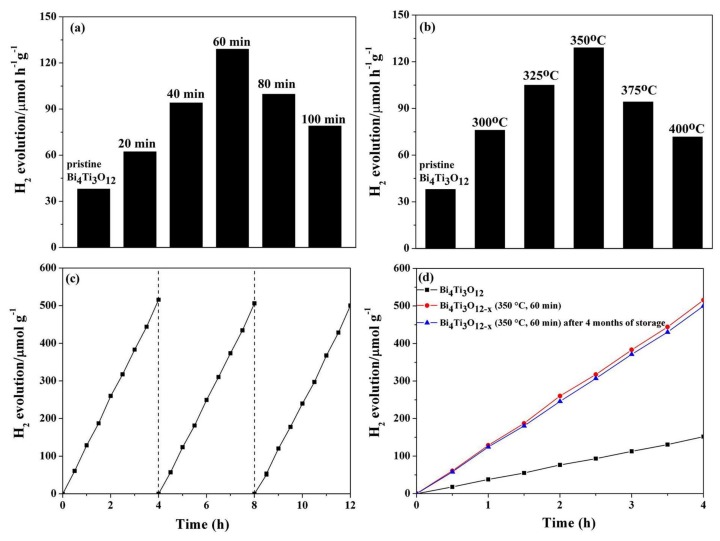
The hydrogen evolution rate over the pristine Bi_4_Ti_3_O_12_ and the Bi_4_Ti_3_O_12−*x*_ after chemical reduction treated (**a**) at 350 °C for various times; (**b**) at various temperature for 60 min under visible-light irradiation (*λ* > 400 nm); (**c**) recycling measure of hydrogen evolution with Bi_4_Ti_3_O_12−*x*_ (350 °C, 60 min) under visible-light irradiation (*λ* > 400 nm); (**d**) visible-light photocatalytic hydrogen evolution by fresh Bi_4_Ti_3_O_12−*x*_ (350 °C, 60 min) and Bi_4_Ti_3_O_12−*x*_ (350 °C, 60 min) after four months of storage, compared with the pristine Bi_4_Ti_3_O_12_.

Photocatalytic conversion of H_2_O into H_2_ using Bi_4_Ti_3_O_12_ or Bi_4_Ti_3_O_12−*x*_ in a methanol-water medium was performed in a quartz cell. Methanol was used to trap holes. Figure 4a,b show the photocatalytic activity of the pristine Bi_4_Ti_3_O_12_ and Bi_4_Ti_3_O_12−*x*_ after chemical reduction treatment at 350 °C for various times and at various temperatures for 60 min for water splitting into H_2_ under visible-light irradiation. It is clear that both the reduction time and temperature have significant influences on the photocatalysis ability of Bi_4_Ti_3_O_12−*x*_. The pristine Bi_4_Ti_3_O_12_ photocatalyst displays the H_2_ evolution rate of around 38 μmol·g^−1^·h^−1^. After the solid-state chemical reduction treatment, the Bi_4_Ti_3_O_12−*x*_ samples all show enhanced photocatalytic activity of the hydrogen evolution. Figure 4a,b show that the photocatalytic activity of Bi_4_Ti_3_O_12−*x*_ improves with both reduction time and temperature increase, until a maximum activity is achieved at 350 °C for 60 min. The H_2_ evolution rate over Bi_4_Ti_3_O_12−*x*_ (350 °C, 60 min) reaches 129 μmol·g^−1^·h^−1^, which is 3.4 times that of the pristine Bi_4_Ti_3_O_12_. This value is also higher than those reported previously (as presented in Table 2). Further increases in the reaction time or temperature results in a reduced hydrogen evolution rate, even though the rates are still higher than when using a pristine Bi_4_Ti_3_O_12_ nanosheet photocatalyst.

To study the reusability and stability of the photocatalyst, cycling experiments using the optimal Bi_4_Ti_3_O_12−*x*_ (350 °C, 60 min) under constant visible-light irradiation were performed. The results obtained from three consecutive experiments are shown in Figure 4c. The first run shows that around 517 μmol·g^−1^ of the total hydrogen evolved after 4 h when Bi_4_Ti_3_O_12−*x*_ (350 °C, 60 min) is used. The second run of the experiment shows a 1.2% decrease in the hydrogen evolution rate comparing to the first run. The hydrogen evolution rate remains almost the same during the third run. The H_2_ evolution rates for the Bi_4_Ti_3_O_12−*x*_ (350 °C, 60 min) photocatalyst remain stable over the three times of cycling testing, confirming good operational stability even after introducing oxygen vacancies into the Bi_4_Ti_3_O_12_ structure. Furthermore, in order to study the long-term stability of Bi_4_Ti_3_O_12−*x*_, the photocatalytic H_2_ production ability of fresh Bi_4_Ti_3_O_12−*x*_ (350 °C, 60 min) and Bi_4_Ti_3_O_12−*x*_ (350 °C, 60 min) after four months of storage were also examined. As shown in Figure 4d, the visible-light photocatalytic activity of the sample stored for four months is only slightly reduced compared with the fresh one, and it is still much higher than that of the pristine Bi_4_Ti_3_O_12_. The results indicate that Bi_4_Ti_3_O_12−*x*_ has good reusability and storage stability. In addition, to measure the apparent quantum efficiency (AQE), the same photocatalytic hydrogen evolution experiment was performed under 420 nm monochromatic lights irradiation, which were obtained by using band-pass filters for 1 h. The AQE was then calculated by the following Equation (5) [40]:

(5) $$\mathrm{AQE}=\frac{N_{e}}{N_{p}}\times 100\%=\frac{2MN_{A}hc}{SPt\lambda }\times 100\%$$

where *N_e_* is the amount of reaction electrons, *N_p_* is the amount of incident photons, *N_A_* is the Avogadro constant, *M* is the amount of H_2_ molecules, *h* is the Planck constant, *c* is the speed of light, *S* is irradiation area, *P* is the average intensity of the irradiation, *t* is the irradiation time, and *λ* is the wavelength of the monochromatic light. For the AQE at 420 nm, the average intensity of the irradiation *P* was determined to be 40 mW/cm^2^, and the irradiation area *S* was 37.5 cm^2^. The calculated AQE for Bi_4_Ti_3_O_12−*x*_ (350 °C, 60 min) is 1.37% under irradiation at 420 nm, which is the highest among all the samples.

In the present work, methanol acts as a sacrificial agent, which is consumed during the formation of H_2_. The photocatalytic mechanisms of methanol-assisted hydrogen evolution are as follows: the electron-hole pairs are produced when a Bi_4_Ti_3_O_12_ or Bi_4_Ti_3_O_12−*x*_ photocatalyst is irradiated with visible light (Equation (6)). The photo-generated carriers either recombine in the bulk or participate in the oxidation-reduction process on the surface of the photocatalyst. In a methanol-water mixture system, methanol can capture photo-generated holes and experience hole oxidation to form formaldehyde (Equation (7)), which reduces the recombination of the electron-hole pairs. Meanwhile, two protons are released during methanol oxidation, which react with the generated electrons to produce H_2_ gas (Equation (8)). When accumulated to a certain degree, formaldehyde is further oxidized into formic acid and releases hydrogen gas (Equations (9) and (10)). The formic acid eventually dissociates into CO_2_ and two protons (Equation (11)); then, the protons react with the photo-generated electrons and produce hydrogen gas (Equation (12)). Equation (13) can be used to represent the overall reaction. The aforementioned photocatalytic hydrogen evolution reactions are summarized below [41,42]:

(6) $${\mathrm{Bi}}_{4}{\mathrm{Ti}}_{3}O_{12}\overset{hv}{\to }h^{+}+e^{-}$$

(7) $${\mathrm{CH}}_{3}\mathrm{OH}+2h^{+}\overset{}{\to }{\mathrm{HCHO}+2[H]}^{+}$$

(8) $${2[H]}^{+}+2e^{-}\overset{}{\to }H_{2}(g)$$

(9) $${\mathrm{HCHO}+2h}^{+}+H_{2}O\overset{}{\to }{\mathrm{HCOOH}+2[H]}^{+}$$

(10) $${2[H]}^{+}+2e^{-}\overset{}{\to }H_{2}(g)$$

(11) $${\mathrm{HCOOH}\;+\;2h}^{+}\;\overset{}{\to }{\mathrm{CO}}_{2}{\left(g)\;+\;2[H\right]}^{+}$$

(12) $${2[H]}^{+}+2e^{-}\overset{}{\to }H_{2}(g)$$

Overall:

(13) $${\mathrm{CH}}_{3}{\mathrm{OH}+H}_{2}O\;\overset{hv,{\mathrm{Bi}}_{4}{\mathrm{Ti}}_{3}O_{12}}{\to }{\mathrm{CO}}_{2}{(g)\;+\;3H}_{2}(g)$$

In addition, photocatalytic conversion of H_2_O into H_2_ using the pristine Bi_4_Ti_3_O_12_ and the optimal Bi_4_Ti_3_O_12−*x*_ (350 °C, 60 min) in pure water were also performed in a quartz cell. The results show that when no methanol is used as a sacrificial reagent, Bi_4_Ti_3_O_12−*x*_ (350 °C, 60 min) shows a very low photocatalytic H_2_ evolution rate of 18 μmol·g^−1^·h^−1^ under visible-light irradiation, and the pristine Bi_4_Ti_3_O_12_ exhibits no H_2_ evolution at all. In a pure water system, water can capture photo-generated holes produced from Bi_4_Ti_3_O_12_ (Equation (6)) and experience hole oxidation to form oxygen gas (Equation (14)). Meanwhile, two protons can be released during water oxidation, which react with the photo-generated electrons to generate hydrogen gas (Equation (15)). Equation (16) can be used to represent the overall reaction:

(14) $$H_{2}O+2h^{+}\overset{}{\to }\frac{1}{2}O_{2}{+2[H]}^{+}$$

(15) $${2[H]}^{+}+2e^{-}\overset{}{\to }H_{2}(g)$$

(16) $$H_{2}O\;\overset{hv,{\mathrm{Bi}}_{4}{\mathrm{Ti}}_{3}O_{12}}{\to }\frac{1}{2}O_{2}{(g)\;+\;H}_{2}(g)$$

However, to trigger this reaction, the energy of the absorbed photon must be at least 1.23 eV, which is much higher than the decomposition energy for methanol (0.7 eV, Equation (13)) [43]. It has been demonstrated that without the addition of a sacrificial agent, the water acts as an inefficient electron acceptor and donor [42,43,44]. As a result, the oxygen radicals and protons tend to recombine to form water, leading to limited hydrogen gas production [44].

**Figure 5:**
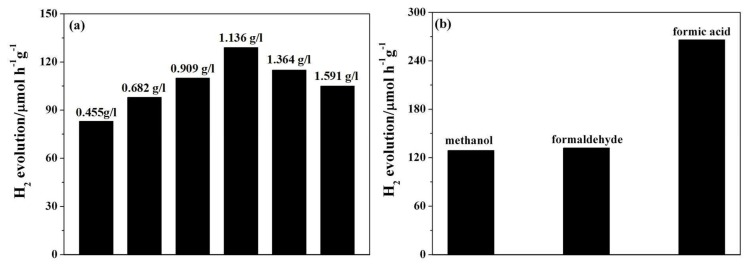
(**a**) Effect of the concentration of the photocatalyst on hydrogen production over the Bi_4_Ti_3_O_12−*x*_ (350 °C, 60 min) nanosheets under visible-light irradiation (*λ* > 400 nm); and (**b**) Effect of various wastes as additives on hydrogen production over the Bi_4_Ti_3_O_12−*x*_ (350 °C, 60 min) nanosheets under visible-light irradiation (*λ* > 400 nm).

The effect of the concentration of the Bi_4_Ti_3_O_12−*x*_ (350 °C, 60 min) photocatalyst on H_2_ production was investigated. Photocatalytic hydrogen evolution experiments were proceeded in a methanol (20 mL)-water (200 mL) mixture. The concentration of the photocatalyst ranged from 0.455 g/L to 1.591 g/L. As shown in Figure 5a, the H_2_ evolution rate increases with the increase of the concentration until a maximum rate is achieved with 1.136 g/L Bi_4_Ti_3_O_12−*x*_ (350 °C, 60 min). Further increase of the concentration results in a reduced hydrogen evolution rate. This reduction may be caused by the unsuited light scattering effect or the light shadowing due to the high turbidity of the solution that reduces the penetration depth of the visible light [45]. These effects reduce the effective incident light, thus significantly reducing the number of photo-induced electron-hole pairs necessary for the maintenance of the reaction. Therefore, the concentration of 1.136 g/L catalyst (i.e., 0.25 g Bi_4_Ti_3_O_12−*x*_ (350 °C, 60 min)) is found to be the optimal concentration for H_2_ generation in the present work.

In addition, the influence of additives, such as formaldehyde and formic acid, on H_2_ production was further investigated. These two kinds of additives are considered mainly because they are byproducts/intermediates of methanol conversion (see Equations (7) and (9)) and also considered to be industrial wastes or model pollutants [46]. A mixture of water-formaldehyde (200/20, *v*/*v*) or water-formic acid (200/20, *v*/*v*) was used in the experiment. Figure 5b shows how different aqueous mixtures (with water-formaldehyde, water-formic acid, and water-methanol) affect visible-light photocatalytic H_2_ production (*λ* > 400 nm) when 0.25 g of the Bi_4_Ti_3_O_12−*x*_ (350 °C, 60 min) photocatalyst is used. It can be seen that the H_2_ evolution rate for formic acid reaches 218 μmol·g^−1^·h^−1^, which is much higher than that for methanol and formaldehyde. It is assumed that this phenomenon is due to the low dissociation energy (−95.8 kJ·mol^−1^) of formic acid that is much smaller than that of methanol (64.1 kJ·mol^−1^) and formaldehyde (47.8 kJ·mol^−1^) [42,47]. Therefore, the –COOH group of formic acid can dissociate spontaneously [42], which results in a large hydrogen evolution rate. The results show that the Bi_4_Ti_3_O_12−*x*_ photocatalyst can be used to decompose a variety of pollutants (such as formaldehyde and formic acid) for hydrogen production, and it also suggests a significant way to produce hydrogen by using formic acid as an additive.

### 3.3. Surface Oxygen Vacancy Formation

**Figure 6:**
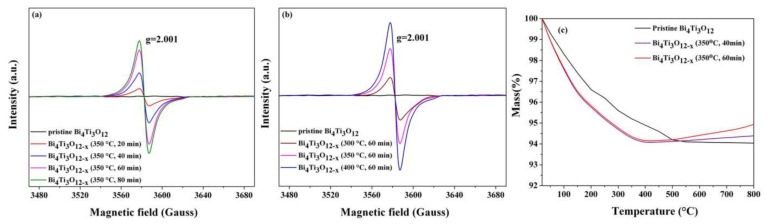
(**a**) electron paramagnetic resonance (EPR) spectra of pristine Bi_4_Ti_3_O_12_ and Bi_4_Ti_3_O_12−*x*_ after chemical reduction treatment (**a**) at 350 °C for different times; (**b**) at different temperatures for 60 min; and (**c**) thermogravimetric analysis (TGA) curves of the pristine Bi_4_Ti_3_O_12_, Bi_4_Ti_3_O_12−*x*_ (350 °C, 40 min), and Bi_4_Ti_3_O_12−*x*_ (350 °C, 60 min).

As discussed above, it is highly probable that the color change of Bi_4_Ti_3_O_12−*x*_ (Figure 2a) could be caused by the formation of oxygen vacancies occurring during the chemical reduction process. To study the presence of oxygen vacancies, room temperature EPR was performed on the pristine Bi_4_Ti_3_O_12_ nanosheets and various Bi_4_Ti_3_O_12−*x*_ samples. It is known that EPR is a highly sensitive and immediate way to characterize oxygen defects [48,49]. As shown in Figure 6a,b, the intensity of the EPR signal at g factor = 2.001 for Bi_4_Ti_3_O_12−*x*_ are all higher than for the pristine Bi_4_Ti_3_O_12_ nanosheets. Typically, a peak at 2.001~2.004 is attributed to natural surface oxygen vacancies as reported in the literature [50,51]. We attribute the enhancement of the EPR signal for Bi_4_Ti_3_O_12−*x*_ at g = 2.001 to the electron-trapped center located around the site of the oxygen vacancies [52]. In addition, it can be seen from Figure 6a,b that the signal intensity at g ~ 2.001 increases with the reduction time and temperature, demonstrating that the number of oxygen vacancies in Bi_4_Ti_3_O_12−*x*_ increases with the reduction reaction time and temperature.

The existence of oxygen vacancies was also proven by TGA testing in the air atmosphere. As shown in Figure 6c, the mass of the pristine Bi_4_Ti_3_O_12_ decreases as the temperature increases because of the desorption of hydroxyl groups physically adsorbed on the surface [53]. When the temperature is above 510 °C, the mass of the pristine Bi_4_Ti_3_O_12_ remains constant. While, as for Bi_4_Ti_3_O_12−*x*_ (350 °C, 60 min), one can notice that when the temperature is below 405 °C, the variation trend of the TGA curve is the same as that of the pristine Bi_4_Ti_3_O_12_. However, when the temperature exceeds 405 °C, there is a clear difference between the mass loss of the pristine Bi_4_Ti_3_O_12_ and Bi_4_Ti_3_O_12−*x*_ (350 °C, 60 min). A slight increase in the mass of Bi_4_Ti_3_O_12−*x*_ (350 °C, 60 min) is observed, which is finished at 800 °C. The same phenomenon was also observed by Li et al. [54] and Yang et al. [55]. It was previously reported by Li et al. that an obvious mass difference between black TiO_2−*x*_ and white TiO_2_ existed during TGA testing. A mass gain for black TiO_2−*x*_ was assigned to the oxidation of oxygen vacancies on the surface [54]. Yang et al. also reported an obvious difference in weight loss between TiO_2_-SO (TiO_2_ with surface oxygen vacancies) and conventional TiO_2_ when the temperature exceeded 440 °C during TGA testing [55]. It was deduced that when the TiO_2_-SO sample was heated in air, its surface oxygen vacancies can be compensated by the external oxygen, resulting in the mass increase for TiO_2_-SO. Therefore, it is postulated that the slight increase in the mass of Bi_4_Ti_3_O_12−*x*_ (350 °C, 60 min) can be explained by the formation of oxygen vacancies on the surface of Bi_4_Ti_3_O_12−*x*_ (350 °C, 60 min) in the present work. When Bi_4_Ti_3_O_12−*x*_ (350 °C, 60 min) with oxygen vacancies is heated in air, its unsaturated surface will be compensated by the external oxygen, leading to a mass increase. In addition, as shown in Figure 6c, the variation trend of the TGA curve of Bi_4_Ti_3_O_12−*x*_ (350 °C, 40 min) is the same as that of Bi_4_Ti_3_O_12−*x*_ (350 °C, 60 min). However, the mass increase of the former is smaller than that of the latter, which may be due to the smaller number of surface oxygen vacancies of Bi_4_Ti_3_O_12−*x*_ (350 °C, 40 min).

**Figure 7:**
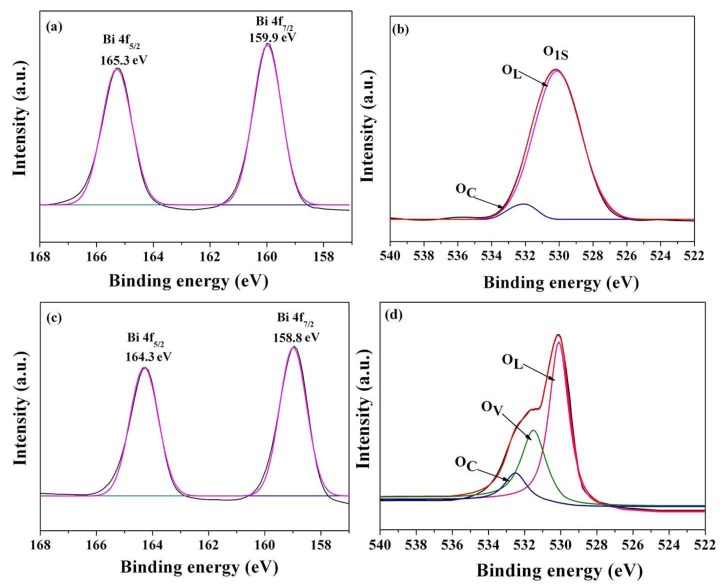
High-resolution X-ray photoelectron spectrometer (XPS) spectra: (**a**) Bi 4f and (**b**) O1s of the pristine Bi_4_Ti_3_O_12_; (**c**) Bi 4f and (**d**) O1s of Bi_4_Ti_3_O_12−*x*_ (350 °C, 60 min).

XPS can provide useful information on the chemical states of elements and surface defects [56]. Figure 7 shows the high-resolution Bi 4f and O1s spectra of the pristine Bi_4_Ti_3_O_12_ and the optimal Bi_4_Ti_3_O_12−*x*_ (350 °C, 60 min). As shown in Figure 7a, the Bi 4f spectrum of the pristine Bi_4_Ti_3_O_12_ sample exhibits two main peaks at 159.9 eV (Bi 4f_7/2_) and 165.3 eV (Bi 4f_5/2_) ascribed to Bi^3+^, which are in accordance with the reported values of Bi_2_O_3_ powders [57,58]. Figure 7b reveals the fitted O1s spectra, where the peaks correspond to the lattice oxygen (O_L_, 530.1 eV) and chemisorbed oxygen species (O_C_, 532.4 eV) on the pristine Bi_4_Ti_3_O_12_ sample, respectively. The oxygen vacancies (O_V_) peak, which should appear at 531.5 eV is not observed in this spectrum, further revealing the stoichiometric properties of the pristine Bi_4_Ti_3_O_12_ sample [59,60]. Figure 7c,d show the high-resolution XPS spectra of the Bi 4f and O1s core levels for the optimal Bi_4_Ti_3_O_12−*x*_ (350 °C, 60 min). The Bi 4f spectrum shows two main peaks centered at 158.8 and 164.3 eV, which are identified as the Bi 4f_7/2_ and Bi 4f_5/2_, respectively. However, 4f_7/2_ and 4f_5/2_ peaks of the metallic Bi are located at 156.8 and 162.2 eV [57]. The chemical shift of the Bi 4f doublet relative to the metallic Bi is about 2.1 eV, which is smaller than the reported value of 3.1 eV between Bi_2_O_3_ and the metallic Bi [61]. This result indicates that the valence state of bismuth in the optimal Bi_4_Ti_3_O_12−*x*_ (350 °C, 60 min) should be (+3 − x) owing to an increased concentration of oxygen defects in the vicinity of Bi ions, which are probably in the Bi_2_O_2_ layer [61]. The XPS spectrum of O1s of the optimal Bi_4_Ti_3_O_12−*x*_ (350 °C, 60 min) is shown in Figure 7d. The O1s XPS spectrum is broad and unsymmetrical, indicating more than one chemical state for oxygen in the optimal Bi_4_Ti_3_O_12−*x*_ (350 °C, 60 min) sample. Gaussian divided features at 530.1 eV, 531.5 eV, and 532.4 eV are credited to the lattice oxygen, oxygen vacancies, and surface chemisorbed oxygen, respectively [59,60]. The O_V_ peak appearing at 531.5 eV indicates that oxygen vacancies are generated in the optimal Bi_4_Ti_3_O_12−*x*_ (350 °C, 60 min) during the solid-state chemical reduction process.

**Figure 8:**
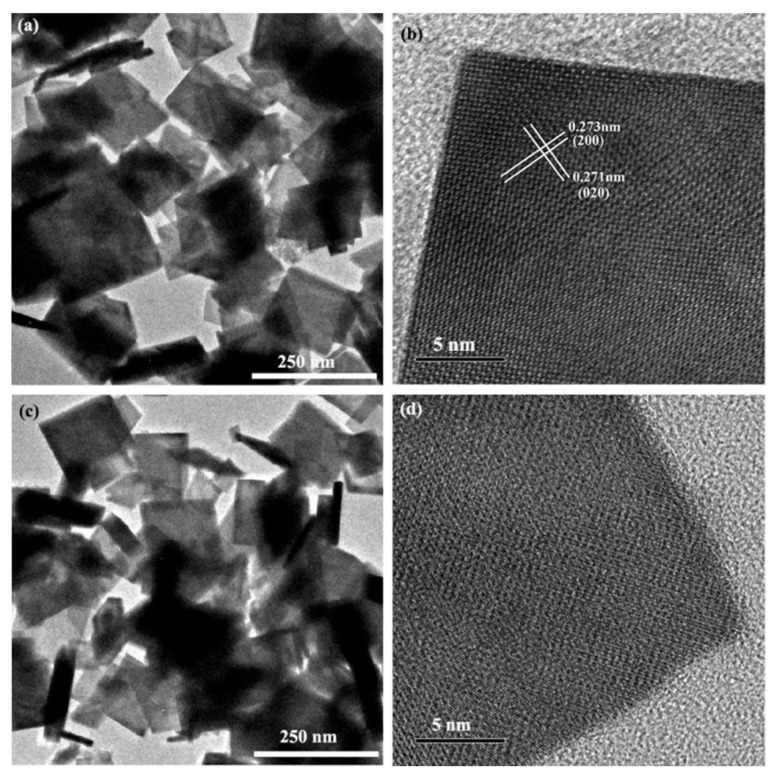
Transmission electron microscopy (TEM) images of (**a**) the pristine Bi_4_Ti_3_O_12_ and (**c**) Bi_4_Ti_3_O_12−*x*_ (350 °C, 60 min); HRTEM images of (**b**) the pristine Bi_4_Ti_3_O_12_ and (**d**) Bi_4_Ti_3_O_12−*x*_ (350 °C, 60 min).

In addition, the change in morphology of the pristine Bi_4_Ti_3_O_12_ and Bi_4_Ti_3_O_12−*x*_ were also scrutinized to prove the formation of oxygen vacancies. Figure 8a,c show representative TEM images of both the pristine Bi_4_Ti_3_O_12_ and the optimal Bi_4_Ti_3_O_12−*x*_ (350 °C, 60 min) photocatalyst, respectively. It is seen that both samples consist of rectangular nanosheets with sides around ~100 and ~150 nm. The particle size of Bi_4_Ti_3_O_12−*x*_ (350 °C, 60 min) has not changed after the solid-state chemical reduction process. HRTEM micrographs offer a more complete view of the microstructures of the samples. As shown in Figure 8b, the pristine Bi_4_Ti_3_O_12_ nanocrystals display a highly crystalline composition, as well as perfect lattice structures throughout the entire particles. The measured spacings are equal to 0.271 nm and to 0.273 nm, which are in agreement with the (020) and (200) planes of Bi_4_Ti_3_O_12_, respectively [62]. However, after the solid-state reduction reaction process at 350 °C for 60 min, a disordered layer is observed on the surface of the Bi_4_Ti_3_O_12−*x*_ (350 °C, 60 min) nanosheet (Figure 8d). Compared with the pristine Bi_4_Ti_3_O_12_ nanocrystals, the lattice features shown in the HRTEM image of Bi_4_Ti_3_O_12−*x*_ (350 °C, 60 min) became highly blurred. The surface structure of the Bi_4_Ti_3_O_12−*x*_ (350 °C, 60 min) nanosheet is imperfect, which may have been damaged by the reduction reaction induced oxygen vacancies [33]. In summary, the EPR, XPS, TGA, and TEM results confirm the existence of oxygen vacancies on the Bi_4_Ti_3_O_12−*x*_ nanosheets, which can be attributed to the reduction of the active hydrogen produced by the decomposition of NaBH_4_.

The concentration and species of the oxygen vacancies in the Bi_4_Ti_3_O_12−*x*_ nanosheets were studied using the positron annihilation life technique [55,63,64,65,66]. Lifetime components (*τ*_1_, *τ*_2_, and *τ*_3_), as well as corresponding intensities (*I*_1_, *I*_2_, and *I*_3_) for the pristine Bi_4_Ti_3_O_12_ and the Bi_4_Ti_3_O_12−*x*_ samples are shown in Table 3. The longest component (*τ*_3_) is typically ascribed to the annihilation of the orthopositronium atom in the material voids [63], and the shortest one (*τ*_1_) is typically due to the annihilation of the positron in the small defects in the bulk, such as the bulk oxygen vacancies [55,64]. Another component (*τ*_2_) arises from positrons trapped by larger-sized defects on the surface of the materials, such as surface oxygen vacancies [55,65]. The relative intensity (*I*_1_/*I*_2_) reflects the ratio of the corresponding defects [55,63] and in the present work, reflects the relative concentration ratio of bulk and surface oxygen vacancies [55]. As shown in Table 3, when the Bi_4_Ti_3_O_12−*x*_ samples were exposed to solid-state chemical reduction treatment at 350 °C, the *I*_1_/*I*_2_ ratio decreased with the increasing chemical reduction reaction time and reached a minimum at 60 min. With further increases in the reaction time and reaction temperature, the *I*_1_/*I*_2_ ratio increased instead. The results of the positron annihilation analysis indicate that the concentrations and types of oxygen vacancies can be controlled by adjusting the chemical reaction time and temperature. This means that due to the presence of active hydrogen produced from the decomposition of NaBH_4_, Bi_4_Ti_3_O_12−*x*_ nanosheets with different reduction degrees can be obtained by tuning the reduction reaction conditions.

### 3.4. Mechanism of Enhanced Photocatalytic Activity of Bi_4_Ti_3_O_12−x_

The photocatalyst’s BET specific surface area was measured to examine a correlation between the surface area and the photocatalytic activity. This is because the large surface areas could influence the number of available active sites [67] and affect the interfacial charge transfer quantum efficiency [68]. As outlined in Table 4, the BET specific surface areas for the Bi_4_Ti_3_O_12−*x*_ samples are almost indistinguishable from the pristine Bi_4_Ti_3_O_12_. In addition, as described earlier, the crystal phase structures are not changed (Figure 3). These results strongly suggest that it is not the surface area or structural features that lead to the large divergence in photocatalysis ability. Therefore, this implies that the photocatalytic kinetics of the Bi_4_Ti_3_O_12−*x*_ samples are mainly enhanced by other factors.

**Figure 9:**
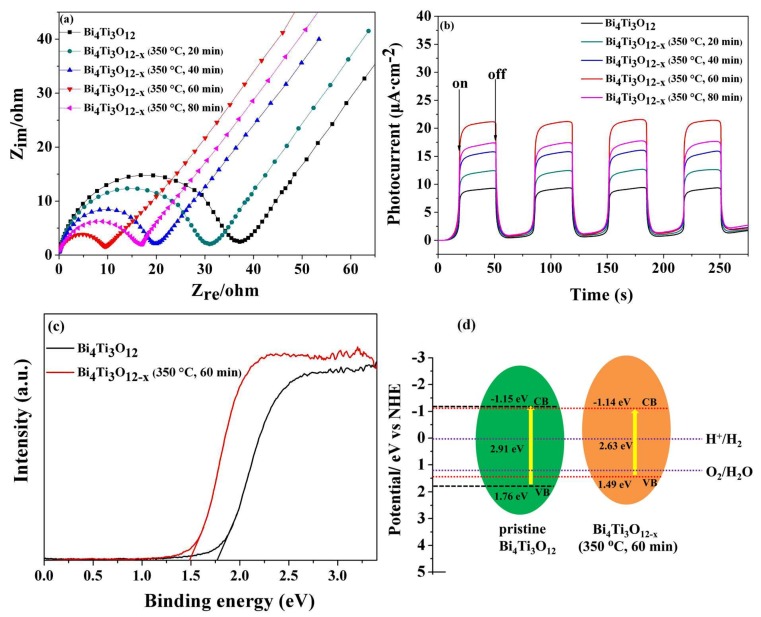
(**a**) The electrochemical impedance spectroscopy (EIS) Nyquist plots of the pristine Bi_4_Ti_3_O_12_ and various Bi_4_Ti_3_O_12−*x*_ samples after the buildup on the ITO electrodes with visible-light (*λ* > 400 nm) irradiation; (**b**) Photocurrents of the pristine Bi_4_Ti_3_O_12_ and various Bi_4_Ti_3_O_12−*x*_ samples after the buildup on the ITO electrodes under visible-light irradiation (*λ* > 400 nm); (**c**) valence band XPS spectra of the pristine Bi_4_Ti_3_O_12_ and Bi_4_Ti_3_O_12−*x*_ (350 °C, 60 min); (**d**) the probable band energy diagram of the pristine Bi_4_Ti_3_O_12_ and Bi_4_Ti_3_O_12−*x*_ (350 °C, 60 min).

As is well-known, generation and disassociation of photo-generated electron-hole pairs are crucial for a semiconductor photocatalyst. The efficiency of this disassociation is central to the enhancement of photocatalytic activity. EIS was used to fully probe this efficiency. Figure 9a shows the EIS of the pristine Bi_4_Ti_3_O_12_ and various Bi_4_Ti_3_O_12−*x*_ electrodes. Each sample diagram contains a semi-circular section, reflecting the process of the charge transfer, as well as a linear section with a 45° slope corresponding to the diffusion-controlled step [69]. The value for the electron-transfer resistance (*R*_ct_) is obtained by calculating the diameter of the semi-circle, and this acts as a proxy for the system’s charge transfer effectiveness. In other words, a smaller *R*_ct_ value means a higher charge transfer efficiency of the system [70]. The order in the *R*_ct_ value is the pristine Bi_4_Ti_3_O_12_ > Bi_4_Ti_3_O_12−*x*_ (350 °C, 40 min) > Bi_4_Ti_3_O_12−*x*_ (350 °C, 80 min) > Bi_4_Ti_3_O_12−*x*_ (350 °C, 60 min), coinciding with the increased activity order of the photocatalysts. The *R*_ct_ of the Bi_4_Ti_3_O_12−*x*_ (350 °C, 60 min) electrode is the smallest among all the catalysts. Therefore, the photo-generated electron-hole pairs are most easily separated and transferred to the surface in the Bi_4_Ti_3_O_12−*x*_ (350 °C, 60 min) sample, thus leading to the highest photocatalytic activity of all the catalysts. The photocurrent analysis was also conducted to confirm the hindering efficiency of Bi_4_Ti_3_O_12−*x*_ during the recombination of electron-hole pairs. Figure 9b details the photocurrent responses of the pristine Bi_4_Ti_3_O_12_, Bi_4_Ti_3_O_12−*x*_ (350 °C, 40 min), Bi_4_Ti_3_O_12−*x*_ (350 °C, 60 min), and Bi_4_Ti_3_O_12−*x*_ (350 °C, 80 min) after their deposition on ITO electrodes under visible light. The results show that the responses are prompt, uniform, and reproducible with the light irradiation switched on and off. Under visible light, the photocurrent density of the Bi_4_Ti_3_O_12−*x*_ (350 °C, 60 min) electrode is the highest among the samples. The enhanced photocurrent indicates the amplification of the photo-induced carrier transport rate, as well as the dwindling photo-generated electron-hole pair recombination rate [71]. The results of the photocurrent investigation are in agreement with the changes in catalytic activity for the pristine Bi_4_Ti_3_O_12−*x*_ and Bi_4_Ti_3_O_12−*x*_ (Figure 4). Therefore, we believe that the improved charge separation and transportation are the major reasons for the enhanced photocatalytic activity of Bi_4_Ti_3_O_12−*x*_.

The position of the valence band (VB) on top of the pristine Bi_4_Ti_3_O_12_ and Bi_4_Ti_3_O_12−*x*_ (350 °C, 60 min) was determined by VB XPS spectra (see Figure 9c). The top of the valence band (E_VB_) of the pristine Bi_4_Ti_3_O_12_ and Bi_4_Ti_3_O_12−*x*_ (350 °C, 60 min) vs. the normal hydrogen electrode (NHE) are estimated to be 1.76 and 1.49 eV, respectively. Moreover, the band gaps of the pristine Bi_4_Ti_3_O_12_ and Bi_4_Ti_3_O_12−*x*_ (350 °C, 60 min) are 2.91 and 2.63 eV, respectively (Table 1). Therefore, using the formula E_CB_ = E_VB_ − Eg [16], the bottom of conduction band (E_CB_) is −1.15 and −1.14 eV for the pristine Bi_4_Ti_3_O_12_ and Bi_4_Ti_3_O_12−*x*_ (350 °C, 60 min), respectively. According to the values of E_VB_ and E_CB_, a suggested band energy diagram is illustrated in Figure 9d. It can be observed that the conduction band energy of Bi_4_Ti_3_O_12−*x*_ (350 °C, 60 min) is almost the same as that of the pristine Bi_4_Ti_3_O_12_. However, compared to the pristine Bi_4_Ti_3_O_12_, the VBM of Bi_4_Ti_3_O_12−*x*_ (350 °C, 60 min) rises considerably, leading to a narrowing band gap of Bi_4_Ti_3_O_12−*x*_ (350 °C, 60 min). We attribute this rise in band gap to the formation of new energy states near the VB top because of the presence of oxygen vacancy in the Bi_4_Ti_3_O_12−*x*_ (350 °C, 60 min) sample [72].

**Figure 10:**
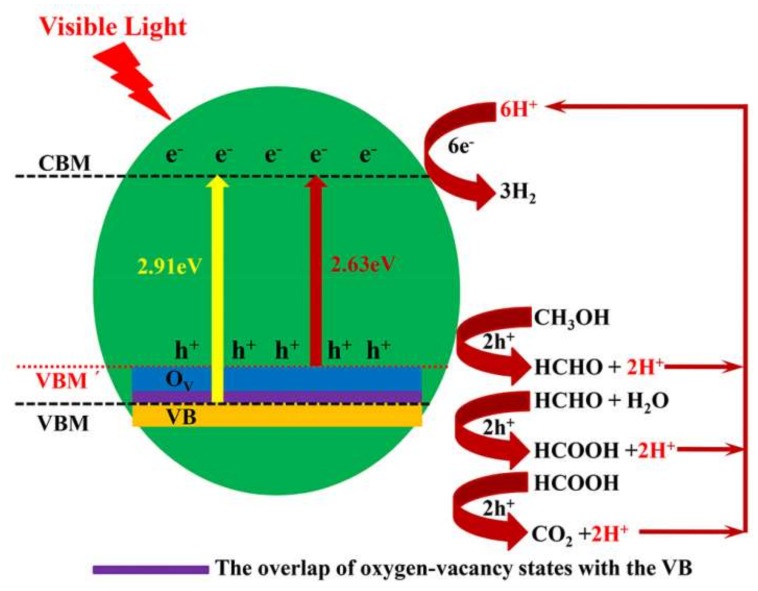
Schematic diagram illustrating the mechanism of the charge separation and photocatalytic reaction for the Bi_4_Ti_3_O_12−*x*_ photocatalyst under visible-light irradiation. VB: valence band; CBM: conduction band minimum; and VBM: valence band maximum.

Based on the discussion above, the reasons for the better photocatalytic activity of the Bi_4_Ti_3_O_12−*x*_ nanosheets could be explained from the point of view of surface defects. The surface oxygen vacancies are located on the top of the VBM or below the conduction band minimum (CBM) [24,48,49,72] and are considered as shallow defects. Zhu et al. discovered that the surface oxygen defect states were formed on the top of the VBM for ZnO and BiPO_4_ [48,49]. Huang et al. demonstrated that a high number of oxygen vacancies created an impurity energy level near the valence band and caused a decrease in the band gap [24]. Zhao et al. reported that under poor oxygen conditions, the oxygen vacancy states at the top of the valence band decreased the band gap of LiTi_2_(PO_4_)_3_ significantly [73]. The rise of the VBM and the reduction of the band of anatase TiO_2_ have been observed by scanning tunneling microscopy [74]. In the present work, the EPR, TGA, and TEM confirm that oxygen vacancies are formed in Bi_4_Ti_3_O_12−*x*_ after the chemical reduction treatment. Figure 10 shows the schematic diagram of the charge separation and photocatalytic reaction for the Bi_4_Ti_3_O_12−*x*_ photocatalyst under visible-light irradiation. Many shallow surface oxygen vacancy states should be above the valence band and partially overlap with the valence band of Bi_4_Ti_3_O_12−*x*_, which can cause rise of the VBM to VBM´. Hence, the VBM of Bi_4_Ti_3_O_12−*x*_ (350 °C, 60 min) is higher than that of the pristine Bi_4_Ti_3_O_12_, which is proven by the valence band XPS spectra (Figure 9c). Correspondingly, the rise of the VBM can further expand the valence band, which can increase the transport rate of photo-generated carriers, resulting in the improved separation efficiency of the photo-generated electron-hole pairs and leading to an obvious improvement of the photocatalytic activities of Bi_4_Ti_3_O_12−*x*_. In addition, due to the VBM’ rise, the band gap of Bi_4_Ti_3_O_12−*x*_ narrows, thus expanding the photoresponse range of Bi_4_Ti_3_O_12−*x*_ (from under 420 nm for the pristine Bi_4_Ti_3_O_12_ to above 460 nm for Bi_4_Ti_3_O_12−*x*_ (350 °C, 60 min)).

Furthermore, as shown in Figure 4, both the reduction time and temperature have significant influence on the photocatalysis ability of the Bi_4_Ti_3_O_12−*x*_ photocatalysts. As discussed above, the positron annihilation analysis (Table 3) indicated that Bi_4_Ti_3_O_12−*x*_ nanosheets with different concentrations and types of oxygen vacancies can be obtained by tuning the conditions of the solid-state chemical reduction process. The relative intensity (*I*_1_/*I*_2_) reflects the relative concentration ratios of bulk and surface oxygen vacancies [55]. When Bi_4_Ti_3_O_12−*x*_ is exposed to chemical reduction treatment at a low temperature or for a short time, the *I*_1_/*I*_2_ ratio continues to decrease with the increasing reaction time and temperature (Table 3), thus increasing the number of surface oxygen vacancies. These surface oxygen vacancies are located above the VB and even partially overlap with it. At the same time, the photocatalytic activity improves gradually with the duration of the treatment and temperature, until a maximum is achieved at 350 °C for 60 min. With additional increases in temperature, as well as prolonged (or longer) reaction time, the *I*_1_/*I*_2_ ratio increases instead (Table 3), which means that the bulk oxygen vacancies continue to increase in Bi_4_Ti_3_O_12−*x*_. The bulk oxygen vacancies’ defect levels form easily in the forbidden band and provide a position for the recombination of the electron-hole pairs, thus reducing the photocatalytic activity [63]. Therefore, the best activity of the Bi_4_Ti_3_O_12−*x*_ photocatalyst can be achieved when the minimum bulk oxygen vacancies exist simultaneously with large numbers of surface oxygen vacancies. Thus, not only the number but also the types of oxygen vacancies induced with varying chemical reduction temperature and duration are essential for a catalyst with high photoactivity.

## 4. Conclusions

In summary, a facile, economic solid-state chemical reduction method has been proposed to fabricate the Bi_4_Ti_3_O_12−*x*_ photocatalyst with abundant oxygen vacancies. The concentration and types of oxygen vacancies could be adjusted by changing the reduction reaction time and temperature. The Bi_4_Ti_3_O_12−*x*_ catalyst showed significantly improved photoactivity during visible-light driven hydrogen evolution from water compared to the pristine Bi_4_Ti_3_O_12_. The hydrogen production rate reaches up to 129 μmol·g^−1^·h^−1^ under visible-light irradiation for the optimal Bi_4_Ti_3_O_12−*x*_ photocatalyst (reduction treated at 350 °C for 60 min), which is about 3.4 times that of the pristine Bi_4_Ti_3_O_12_. It is proposed that energy levels corresponding to the surface oxygen vacancies should be above and partially overlap with the Bi_4_Ti_3_O_12−*x*_ valence band. This can raise the top of the valence band maximum. The improved photoactivity of the photocatalyst is the result of the enhanced separation ability of photo-generated electron-hole pairs that originates from the valence band expansion by the surface oxygen vacancy states. The extended photoresponse is due to the decrease in the band gap caused by the rise of the top of the valence band maximum.

## Figures and Tables

**Table 1:** Band gaps of the pristine Bi_4_Ti_3_O_12_ and various Bi_4_Ti_3_O_12−*x*_ samples.

Samples	Band Gap (eV)
Bi_4_Ti_3_O_12_	2.91
Bi_4_Ti_3_O_12−*x*_ (350 °C, 20 min)	2.83
Bi_4_Ti_3_O_12−*x*_ (350 °C, 40 min)	2.74
Bi_4_Ti_3_O_12−*x*_ (350 °C, 60 min)	2.63
Bi_4_Ti_3_O_12−*x*_ (350 °C, 80 min)	2.57
Bi_4_Ti_3_O_12−*x*_ (350 °C, 100 min)	2.48
Bi_4_Ti_3_O_12−*x*_ (300 °C, 60 min)	2.77
Bi_4_Ti_3_O_12−*x*_ (400 °C, 60 min)	2.39

**Table 2:** Comparison of H_2_ evolution rate of Bi_4_Ti_3_O_12−*x*_ (350 °C, 60 min) and other Bi_4_Ti_3_O_12_ photocatalysts recently reported.

Sample	Light Source	Reactant Solution	H_2_ Evolution Rate/μmol·g^−1^·h^−1^	Reference
Bi_4_Ti_3_O_12−*x*_ (350 °C, 60 min)	300 W Xe Lamp (*λ* > 400 nm)	200 mL water + 20 mL methanol	129	This work
Bi_4_Ti_3_O_12_	350 W high pressure Xe lamp (*λ* > 400 nm)	400 mL water + 20 mL methanol	36	[37]
Bi_4_Ti_2.6_Cr_0.4_O_12_	350 W high pressure Xe lamp (*λ* > 400 nm)	400 mL water + 20 mL methanol	58.1	[37]
Bi_4_Ti_3_O_12_	300 W Xe Lamp (*λ* > 400 nm)	400 mL water + 20 mL methanol	42	[38]
Bi_4_Ti_2.6_Cr_0.4_O_12_	300 W Xe Lamp (*λ* > 400 nm)	400 mL water + 20 mL methanol	98	[38]
Bi_4_Ti_2.6_Cr_0.4_O_12_	300 W Xe Lamp (*λ* > 420 nm)	400 mL water + 30 mL methanol	117	[39]

**Table 3:** Positron lifetime and relative intensities of the pristine Bi_4_Ti_3_O_12_ and various Bi_4_Ti_3_O_12−*x*_ samples.

Sample	*τ*_1_ (ps)	*τ*_2_ (ps)	*τ*_3_ (ns)	*I*_1_ (%)	*I*_2_ (%)	*I*_3_ (%)	*I*_1_/*I*_2_
Bi_4_Ti_3_O_12_	193	376	2.33	50.24	47.78	1.98	1.05
Bi_4_Ti_3_O_12−*x*_ (350 °C, 20 min)	196	387	2.47	46.26	51.97	1.77	0.89
Bi_4_Ti_3_O_12−*x*_ (350 °C, 40 min)	199	389	2.49	38.72	59.64	1.64	0.65
Bi_4_Ti_3_O_12−*x*_ (350 °C, 60 min)	205	393	2.77	23.87	74.24	1.89	0.32
Bi_4_Ti_3_O_12−*x*_ (350 °C, 80 min)	209	396	2.92	36.48	61.57	1.95	0.59
Bi_4_Ti_3_O_12−*x*_ (350 °C, 100 min)	214	402	3.05	41.73	56.42	1.85	0.74
Bi_4_Ti_3_O_12−*x*_ (400 °C, 60 min)	216	405	3.11	44.86	53.33	1.81	0.84

**Table 4:** Brunauer-Emmett-Teller (BET) specific surface areas of the pristine Bi_4_Ti_3_O_12_ and various Bi_4_Ti_3_O_12−*x*_ samples.

Samples	BET Specific Surface Area (m^2^/g)
Bi_4_Ti_3_O_12_	6.45
Bi_4_Ti_3_O_12−*x*_ (350 °C, 20 min)	6.39
Bi_4_Ti_3_O_12−*x*_ (350 °C, 40 min)	6.35
Bi_4_Ti_3_O_12−*x*_ (350 °C, 60 min)	6.32
Bi_4_Ti_3_O_12−*x*_ (350 °C, 80 min)	6.46
Bi_4_Ti_3_O_12−*x*_ (350 °C, 100 min)	6.48
Bi_4_Ti_3_O_12−*x*_ (300 °C, 60 min)	6.38
Bi_4_Ti_3_O_12−*x*_ (400 °C, 60 min)	6.51

## Supplementary Materials

The following are available online at http://www.mdpi.com/2079-4991/8/4/261/s1.
