# Supplementary material for: A Facile Method for the Preparation of Colored Bi4Ti3O12−x Nanosheets with Enhanced Visible-Light Photocatalytic Hydrogen Evolution Activity
Source: Nanomaterials (Basel). 2018 Apr 21;8(4):261. doi: 10.3390/nano8040261 (PMC5923591; doi:10.3390/nano8040261)
Supplement: Supplementary file 1 [file nanomaterials-08-00261-s001.pdf]

Supplementary Materials

A Facile Method for the Preparation of Colored $Bi_4Ti_3O_{12-x}$ Nanosheets with Enhanced Visible Light Photocatalytic Hydrogen Evolution Activity

Yizeng Zhang [1], Zhiwu Chen [1,2,*], and Zhenya Lu [1]

Synthesis of $Bi_4Ti_3O_{12}$ Nanosheets

$Bi_4Ti_3O_{12}$ nanosheets were prepared via sol-gel synthesis and subsequent hydrothermal processing. First, according to the composition of $Bi_4Ti_3O_{12}$, solutions A and B were obtained by dissolving 0.02 mol $Bi(NO_3)_3{\cdot}5H_2O$ in 15ml acetic acid and 0.15 mol $Ti(C_4H_9O)_4$ in 15 ml ethylene glycol monomethylether under stirring at room temperature, respectively. All reagents were of analytical grade. Solution B was slowly added into solution A dropwise, and the mixture was vigorously stirred for 2 h, resulting in a homogeneous sol. The sol was stabilized when its concentration was adjusted to 0.1 M by adding 10 ml acetic acid and 10 ml ethylene glycol monomethylether, before being heated to 80 °C for 24 h to produce dry gel. The gel was added to 75 ml of a 3 M NaOH solution to form a suspension, poured into a Teflon-lined, stainless-steel autoclave (volume of 100 ml) with a filling capacity of 80%, and subjected to hydrothermal treatment at 160 °C for 16 h unless otherwise stated. Finally, the synthesized product was cooled down, filtered, washed with distilled water, and dried at room temperature.

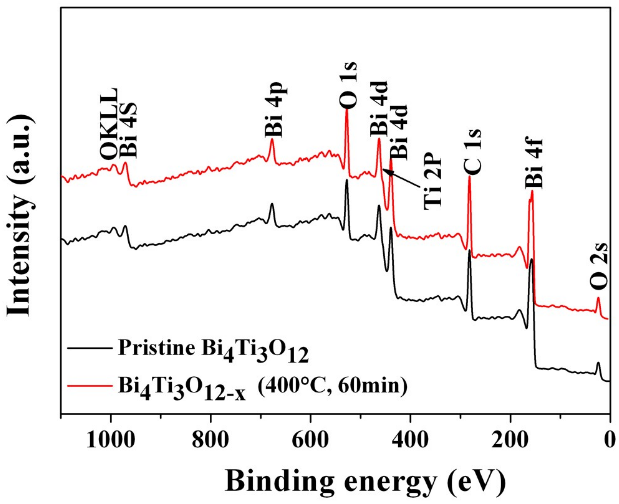

Figure S1. XPS survey spectra for (a) pristine $Bi_4Ti_3O_{12}$ and (b) $Bi_4Ti_3O_{12-x}$ (350 °C, 60 min).

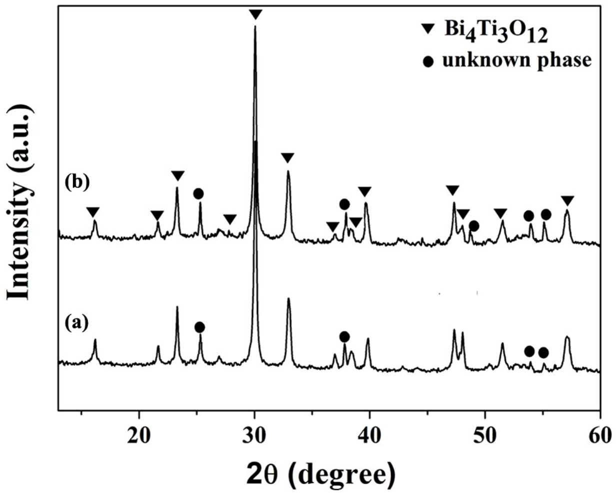

Figure S2. X-ray diffraction patterns of (a) $Bi_4Ti_3O_{12-x}$ (350 °C, 120 min) and (b) $Bi_4Ti_3O_{12-x}$ (400 °C, 80 min).
